# Response Surface Method Analysis of Chemically Stabilized Fiber-Reinforced Soil

**DOI:** 10.3390/ma14061535

**Published:** 2021-03-21

**Authors:** Abdullah Almajed, Dinesh Srirama, Arif Ali Baig Moghal

**Affiliations:** 1 Department of Civil Engineering, College of Civil Engineering, King Saud University, Riyadh 11421, Saudi Arabia; 2 Department of Civil Engineering, National Institute of Technology, Warangal 506004, Telangana State, India; dinesh1991@student.nitw.ac.in (D.S.); or reach2arif@gmail.com (A.A.B.M.)

**Keywords:** response surface methodology, optimization, lime, fiber, stabilization

## Abstract

One of the significant issues persisting in the study of soil stabilization is the establishment of the optimum proportions of the quantity of stabilizer to be added to the soil. Determining optimum solutions or the most feasible remedies for the utilization of stabilizing products in terms of their dose rates has become a significant concern in major civil engineering design projects. Using the response surface methodology, this study primarily focused on investigating the optimal levels of reinforcement fiber length (FL), fiber dosage (FD), and curing time (CT) for geotechnical parameters of stabilized soil. To realize this objective, an experimental study was undertaken on the California bearing ratio (CBR) and unconfined compressive strength (UCS). Hydraulic conductivity (HC) tests were also performed, with stabilizer proportions of 6–12 mm for the FL and 0.2–0.6% for the FD calculated for the total dry weight of soil and 6% lime (total weight of dry soil). The curing times used for testing were 0, 7, and 14 days for the CBR tests; 60, 210, and 360 days for the UCS tests; and 7, 17, and 28 days for the HC tests. All practical experiments were conducted with experimental techniques using stabilizer proportions and curing times. The FL, FD, CT, CBR, UCS, and HC response factors were determined using the central composite design. The results point toward a statistically significant model constructed (*p* ≤ 0.05) using the analysis of variance. The results from this optimization procedure show that the optimal values for the FL, FD, and CT were 11.1 mm, 0.5%, and 13.2 days, respectively, as these provided the maximum values for the CBR; 11.7 mm for the FL, 0.3% for the FD, and 160 days for the CT corresponded to the maximum values for the UCS; and 10.5 mm for the FL, 0.5% for the FD, and 15 days for the CT led to the minimum value for the HC. In practice, the suggested values may be useful for experiments, especially for preliminary assessments prior to stabilization.

## 1. Introduction

Site feasibility is the major hindrance in most civil engineering projects. In the majority of civil engineering projects worldwide, expansive soils have substantial geotechnical and structural design-related issues, with economic difficulties estimated to entail costs of several billion dollars annually [1,2,3]. These soils are usually rich in montmorillonite mineral, which causes significant volume changes (shrink/swell) with variations in moisture content [4,5].

In many instances, naturally available soils require lasting alternatives because they do not meet specific geotechnical properties [6]. Improvement in the various geotechnical properties of soil can be achieved by stabilization with suitable materials. Recent advances have shown a rising interest in chemical and bio-geochemical alterations of soils hat enhance their geotechnical properties [5,7]. Among these methods, lime stabilization is the most sought after technique due to its versatility and innate potential in addressing the distress-related issues of expansive plastic fines [8,9,10,11]. Even though lime usage affects the rapid and significant loss in strength resulting from brittle failure characteristics, many researchers have found it most effective in enhancing expansive soil properties. The past few years have seen growing attention paid to expansive soil reinforcement with different fibers. The reinforcements of various shapes and dimensions are made out of geo-synthetic materials or short fiber strips [12]. A notable increase in the soil index and engineering properties of cohesive soil was achieved after strengthening with discrete polypropylene fiber [13]. Many researchers have studied the effects of polypropylene fiber content, polypropylene fiber length (FL), lime content, and curing time (CT) on the various engineering properties of expansive soil [9,14,15]. When amended with the soil medium, polypropylene fiber materials demonstrate proven functionality in durability and sustain soil index and engineering properties improvements in the long run [14,15].

Fiber Cast (FC) fibers have a better swell-limiting efficiency in the absence of lime treatment [16]. The nature and type of fiber, dosage and length have a considerable impact on the California bearing ratio (CBR) values of natural soils and soils stabilized using lime [17]. Mean FC and Fiber Mesh (FM) dimensions and concentrations are vital in designing the variables of fiber-reinforced lime-amended expansive soils, predominantly affecting the subgrade stability [18]. One of the significant issues persisting in soil stabilization is the establishment of an optimum quantity of stabilizer to be added to the soil. This involves an excessive amount of energy and time for investigation and necessitates an enormous number of experiments. These downsides could be well avoided by determining the optimal or best possible solutions using approximation concepts, mathematical system modeling, or optimization procedures [6,7,8,9,10,11,12,13,14,15]. The inspiration behind the usage of optimization procedures lies in quality improvement and in cost reduction.

Experimentation based on the measurement of one or more responses (variables) plays a significant part in several science and technology areas. Planning and designing experiments, analyzing the results and observing the process and the system operation are necessary to obtain a final result. The response surface methodology (RSM) is one of the most commonly used experimental designs for optimization [19]. It is a compilation of both numerical and statistical methods helpful for building an empirical model and optimization process parameters and is mostly used in finding the interaction of numerous affecting factors [20]. Through the design of experiments, the RSM expels systematic errors and reduces the number of experiments required to obtain the optimum values. It comprises a set of mathematical and analytical methods that are valuable for establishing the building of the empirical design and enhancing and maximizing process specification. The RSM can also be utilized to discover the interaction of numerous impacting aspects [21]. The RSM optimization involves three primary steps: (1) statistically calculated trials; (2) estimating the coefficients; and (3) predicting the response and validating the model adequacy with the experimental arrangement [22].

The present study examined the determination of input variables (i.e., amount and length of fibers and CT) that could potentially offer the optimum values of various geotechnical parameters, such as the unconfined compression strength (UCS), hydraulic conductivity (HC), and California bearing ratio, through optimization using face-centered central composite design (FCCCD) in conjunction with an RSM study of regular expansive semi-arid soil from the municipality of Al-Ghat. The addition of lime (quick lime) was considered to ensure the proper bonding between clay particles and fiber components, with 6% lime dosage [23]. The impacts of various values for the soil fiber dosage (FD) (0.2%, 0.4%, and 0.6% by dry weight), the FL (6, 9, and 12 mm), and the CT (0–14 days for the CBR, 60–360 for the UCS, and 7–28 days for the HC) on the geotechnical parameters studied for the targeted soil using the RSM were evaluated. The optimum values for soil stabilization for various applications are presented.

## 2. Experimental Investigation

### 2.1. Materials

The soil used in this study was obtained from the municipality of Al-Ghat where the soil is known to have distinct mineralogical and plasticity properties. The soil is classified as clay of high plasticity (CH) according to the Unified Soil Classification System (USCS). The maximum dry density and optimum moisture content were 1.64 kN/m^3^ and 23%, respectively. Its plasticity index is 31%. In its natural state, the soil has 3.2% moisture content and had a specific gravity value of 2.85. Analytical grade quick lime (supplied by Winlab Chemicals, UK) was utilized as a chemical additive. Polypropylene fibers (Fiber Cast) acquired from Propex Operating Company (LLC, UK) were used. This fiber has very low electrical conductivity and high acid and salt resistance. The tensile strength was found to be 440 N/mm^2^. The melting and ignition points were found to be 324 °F (162 °C) and 1100 °F (593 °C), respectively. The fiber is alkali-proof and has zero water absorption ability.

### 2.2. Software Used

Design-Expert, released by Stat-Ease Inc., is an open-source statistical software package for building full quadratic models using RSM and performing analysis of variance (ANOVA). It provides powerful tools for designing an ideal experiment in terms of process, mixture, or a combination of factors and components.

### 2.3. Sample Preparation and Experimental Procedure

Lime addition (6% by dry weight of soil) to dry soil was carried out prior to mixing fibers. Close attention was paid to the mixing process to maintain a homogenous mixture [24]. All samples were compacted at their maximum dry density values in accordance with ASTM D698 [25]. Following the proper mixing of the respective fibers for each mix (based on the dry weight of soil), tests were performed following the relevant codes mentioned in Table 1. For hydraulic conductivity tests, the compacted specimen along with a Perspex hydraulic conductivity mold were kept in a desiccator, maintaining a relative humidity of more than 95%, and cured for 7 and 28 days. For the unconfined compression tests, the compacted soil samples wrapped in plastic were preserved in humidity-controlled chambers (maintained at 100% relative humidity) to avoid any moisture movements. The samples were cured for 60 and 360 days, following which the specimen weight was noted. Any sample which recorded a loss of weight of 5% due to heat or hydration was rejected prior to testing. For CBR tests, the force required for a penetration of up to 12.5 mm was determined on the samples cured for 14 days (and also for the immediate case at 0 days of the curing period), which were tested at their respective optimum moisture content and maximum dry density values. For each geotechnical parameter, samples were tested in triplicate. The dispersion of the results was found to be within 5% confidence limits, as per relevant ASTM standards, for each test considered. The experimental CBR values under the soaked condition, the UCS values, the and HC values were determined for respective curing times of 0–14 days for the CBR, 60–360 days for the UCS, and 7–28 days for the HC (Table 2, Table 3 and Table 4, respectively).

### 2.4. Experimental Results

Table 2 reveals that CBR characteristics were significantly increased with curing period. The presence of lime triggered the formation of cementitious compounds, resulting in higher CBR values. Along similar lines, the UCS values increased significantly up to 210 days, after which there was not much of a significant increase in the values. Beyond 210 days of the curing period, the readily available reactive silica was consumed, resulting in a net reduction in the pH of the soil-system, and thus there was no further contribution to any increase in strength. Hence, the rate of gain in the UCS from 210 to 360 days proceeded at a slower pace, as seen from Table 3. On the other hand, the HC values of fiber-treated lime-amended specimens increased with the addition of fiber compared to the untreated case, as seen from Table 4. With an increase in curing period, the HC values were reduced significantly, which can be attributed to the formation of cementitious compounds due to the addition of lime, making the treated soil less conductive. Similar observations have been reported by Moghal et al. [14,15,16,17,18] for fiber-reinforced lime-blended expansive semi-arid soils.

## 3. RSM

### 3.1. Experimental Design

For the experimental design, a face-centered central composite design (FCCCD) was employed to build the RSM model. A three-factor experimental design, consisting of eight factor points representing the upper and lower limits of the three input variables, six axial points representing how far outside the factorial limits a parametric value can be comprehensive, and six center points representing the mean values of the input variables, was created.

### 3.2. Model Development

The inclusion rates of the independent variables were the fiber lengths of 6, 9, and 12 mm, fiber dosages of 0.2, 0.4, and 0.6% by soil dry weight, and CTs of 0–14 days. The FCCCD was employed, which used only three levels, “1,” “0”, and “−1”, representing the maximum, mean, and minimum actual values of the input variables, respectively. Table 5 lists the experimental levels of each variable. A quadratic model with three factors and three levels with 95% confidence levels was employed to evaluate the RSM model. The backward elimination technique was applied with only statistically significant terms to represent the relationship between the input factors and the output response variables (*p* ≤ 0.05). Table 6, Table 7 and Table 8 present the values obtained from the experimental procedure and measured and predicted values from the RSM models of the CBR, UCS, and HC, respectively.

### 3.3. Validation of the Built Model

In the response surface method, the relationships between the independent and dependent variables were determined using multiple regression techniques. The regression equations used to build models to determine the relationships between variables and represent the total effect of all variables obtained from the experimental or actual values had to be validated with the approximation values obtained from the models. It was also essential to verify whether any of the least-squares regression assumptions have been violated. The coefficient (R^2^), adjusted R^2^ (Adj R^2^), predicted R^2^ (Pred R^2^), and adequate precision (AP) values were then computed.

## 4. Results and Discussions

### 4.1. Statistical Assessment of the Experimental Results of the CBR

The influence on the ANOVA dependent variable resulting from different independent factors was evaluated using a parametric statistical technique. The influence of independent factors (i.e., FL, FD, and CT) and their interactions on the response variable (i.e., the CBR value) was studied with ANOVA, which is a useful statistical tool for describing the interactions of variables with each other. As previously mentioned, the statistical significance of independent parameters (i.e., FL, FD, and CT) with regard to the dependent variable (i.e., the CBR value) experimental results at a 95% confidence level was analyzed using a full quadratic model. The statistically insignificant terms, with *p* greater than 0.05, were removed by performing a backward analysis on the full quadratic equation. The percentage contributions to the response parameters in the model built were determined. ANOVA was performed, with the results containing enumerated terms (Table S1).

Table 6 compiles the CBR values obtained through the experiments performed in the lab and measured from the FCCCD experiments designed using Design-Expert software. The following models (Equation (1)) were developed for the CBR values using the regression coefficients summarized in Table S4.

CRB = (8.65646 + (0.251732 × FD) + (5.10504 × FL) + (0.239471 × CT) − (0.250000 × (FD × FL)) + (0.022619 × (FD × CT)) + (0.535714 × (FL × CT)) + (0.036507 × CT^2^) (1)

Table S1 shows *p* < 0.05, which indicates significant model terms.

**Figure 1:**
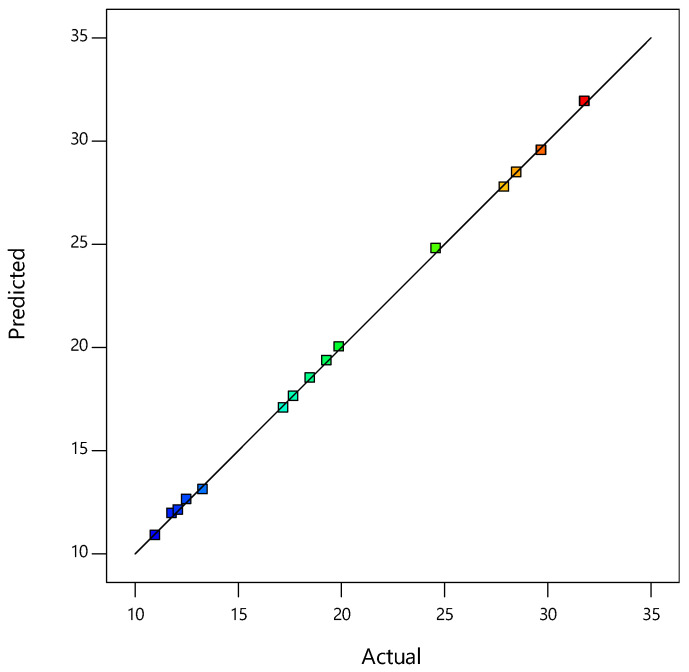
Plot of predicted vs. actual values for the CBR

**Figure 2:**
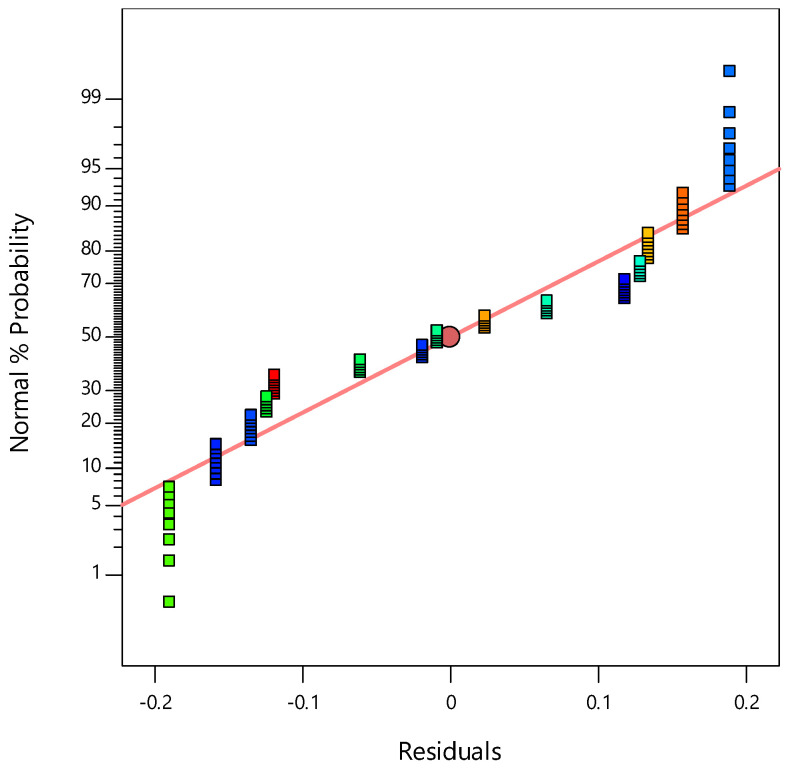
Plot of normal probability of residuals

The model F value of 33,444.15 indicates the model significance. This calculation determines the ratio of explained to unexplained variance. Computations were performed for the statistically significant parameters, whereas insignificant parameters (with *p* > 0.05) were removed using backward analysis from a complete RSM quadratic model. Figure 1 depicts the plot of predicted versus actual values for the CBR and Figure 2 depicts the contributions of each parameter to the constructed RSM model.

The CBR values built using the RSM model and Equation (1) were validated for the R^2^, Adj R^2^, Pred R^2^, and AP statistics (Table 7). With a difference of less than 0.2, the Pred R^2^ of 0.9996 was in reasonable agreement with the Adj R^2^ of 0.9997. As shown in Table S1, the R^2^ of the model was 0.9996 (close to 1), indicating the variation of the response variable through input factors by robust fitting.

Adequate precision is desirable, with a ratio greater than 4. A 510.030 ratio indicates an adequate signal to ensure the possibility of using the model in navigating the design space. The Pred R^2^, explaining the variance in the new data of the constructed model, was 0.9996 (Table S1), indicating that approximately 99.96% of the variability in the estimation of the new response values can be explained by the built RSM model.

Figure 1 shows that the prediction provided by the model equation vs. the experimental values was a statistically good match. Figure 2 displays the average probability of residuals for a response, confirming whether a normal distribution follows the standard deviations between the actual and predicted response values [33,34].

**Figure 3:**
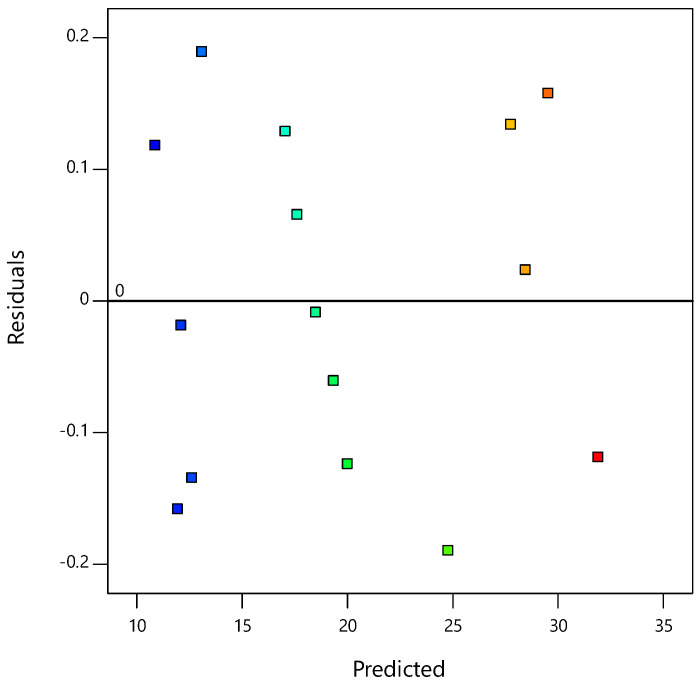
Plot of the residuals vs. the predicted responses for the CBR values

Figure 3 plots the residuals against the predicted response, whereby the constant variance assumption can be verified. All points of the experimental runs were randomly distributed. All values were within the −0.2 to 0.2 range, suggesting that the models proposed by the RSM were satisfactory and confirm the constant variance assumptions.

**Figure 4:**
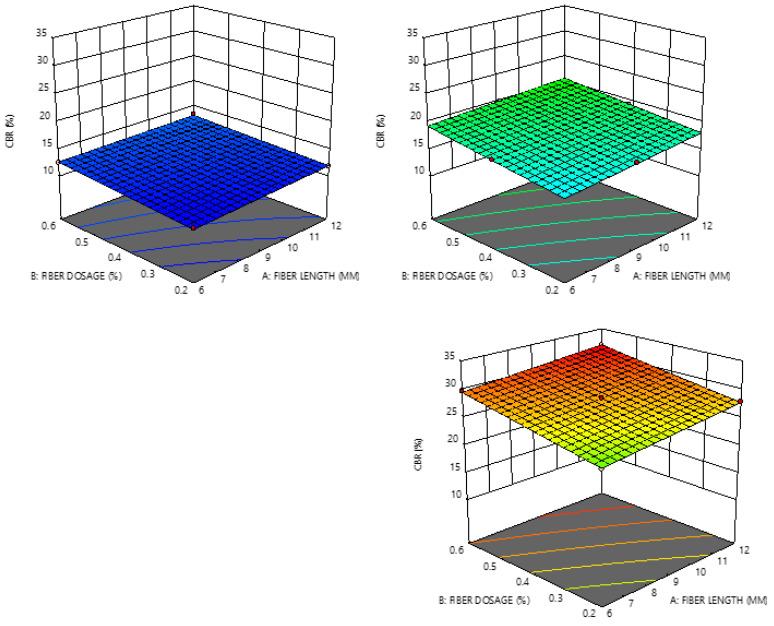
Three-dimensional surface plots showing relationships between responses and variables

The influence of the parameters on the relationship between the dependent and independent variables is visualized by the 3D surface plots in Figure 4. One of the influential parameters was CT, considering the increase in the CBR with the increase in the CT.

### 4.2. Statistical Assessment of the Experimental Results of the UCS

The influence of independent factors (i.e., FL, FD, and CT) and their interactions on the response variable (i.e., the UCS value) was studied with ANOVA. As previously discussed, the statistical significance of independent parameters (i.e., FL, FD, CT) with regard to the dependent variable (the UCS value) experimental results at a 95% confidence level was analyzed using a full quadratic model. The statistically insignificant terms, with p greater than 0.05, were removed by performing backward analysis on the full quadratic equation. The percentage contributions to the response parameters in the model built were obtained. ANOVA was then performed, with the results containing enumerated terms shown in Table S2.

Table 7 presents the UCS values obtained by the experiments performed in the lab and measured from the FCCCD experiments designed using Design-Expert software. The following models (Equation (2)) were developed for the UCS values using the regression coefficient in Table S2.

UCS = 2445.03 + 160.257 × A + 151.905 × C − 189.415 × AB − 80.28 × AC − 84.3816 × BC − 167.996 × C^2^ (2)

**Figure 5:**
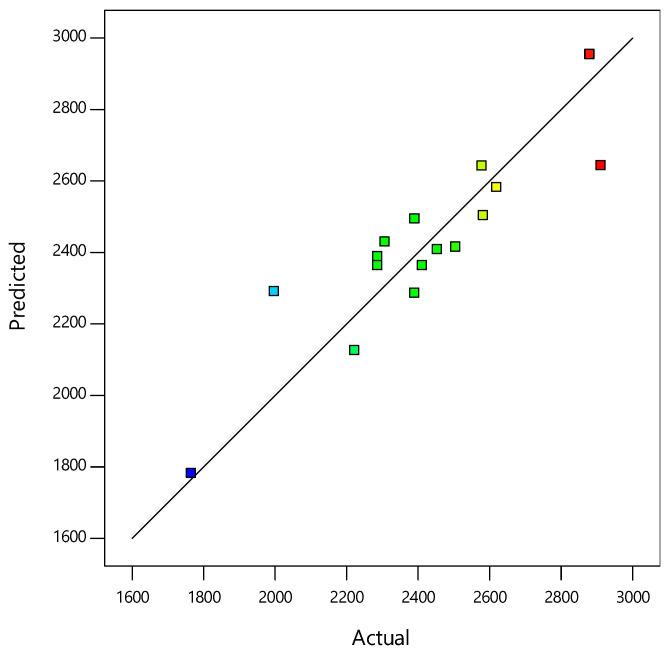
Predicted vs. actual values of the UCS

As mentioned earlier, in Table S2, the A, C, AB, AC, BC, C^2^ *p*-values less than 0.0500 and the model f value of 50.36 indicate that the model terms were significant. The Pred R^2^ of 0.7881 was in reasonable agreement with the Adj R^2^ of 0.8088 (i.e., the difference was less than 0.2). An Adequate precision ratio of 30.037 indicated an adequate signal. The Pred R^2^ of the model was 0.7881 (Table S2). The model adequacy was checked by correlating the observed and predicted data. Figure 5 shows that the model equation prediction experimental values for the UCS were a statistically good match.

**Figure 6:**
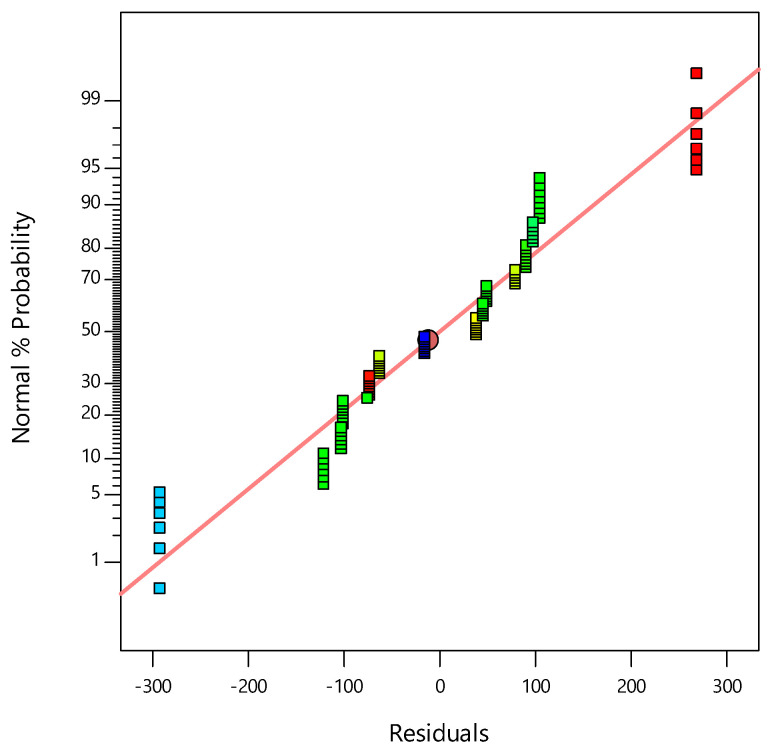
Normal probabilities of the UCS residuals

Figure 6 represents the average probability of the response residuals, indicating that the experimental values were distributed comparatively close to the straight line. A satisfactory correlation was found between these values.

**Figure 7:**
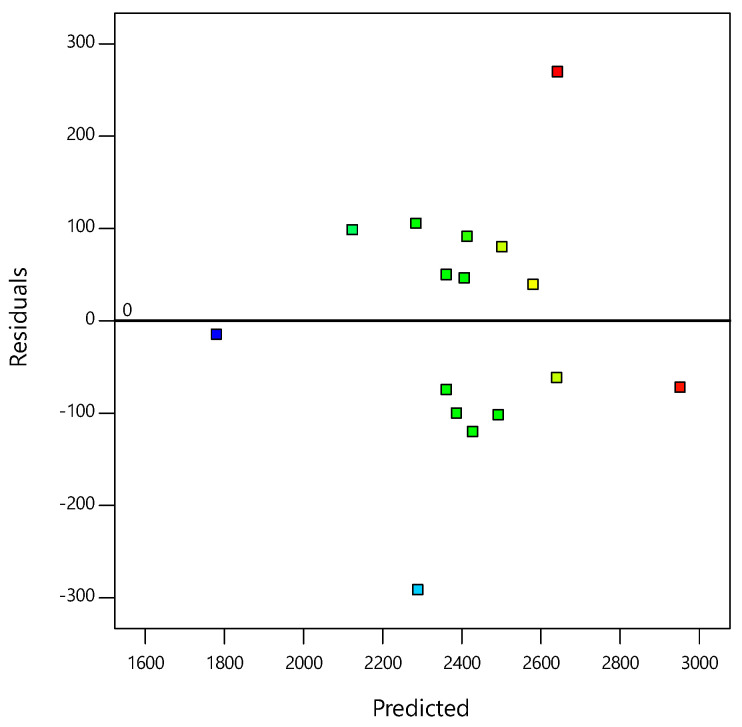
Plot of the residuals vs. predicted responses for the CBR values

The residual plotted against the predicted response verified the constant variance assumption for CBR values as seen from Figure 7. All points of the experimental runs were randomly distributed. All values lay mostly within the range of −150 and 150. These results suggest that the models proposed by the RSM were satisfactory and that the constant variance assumptions were confirmed.

**Figure 8:**
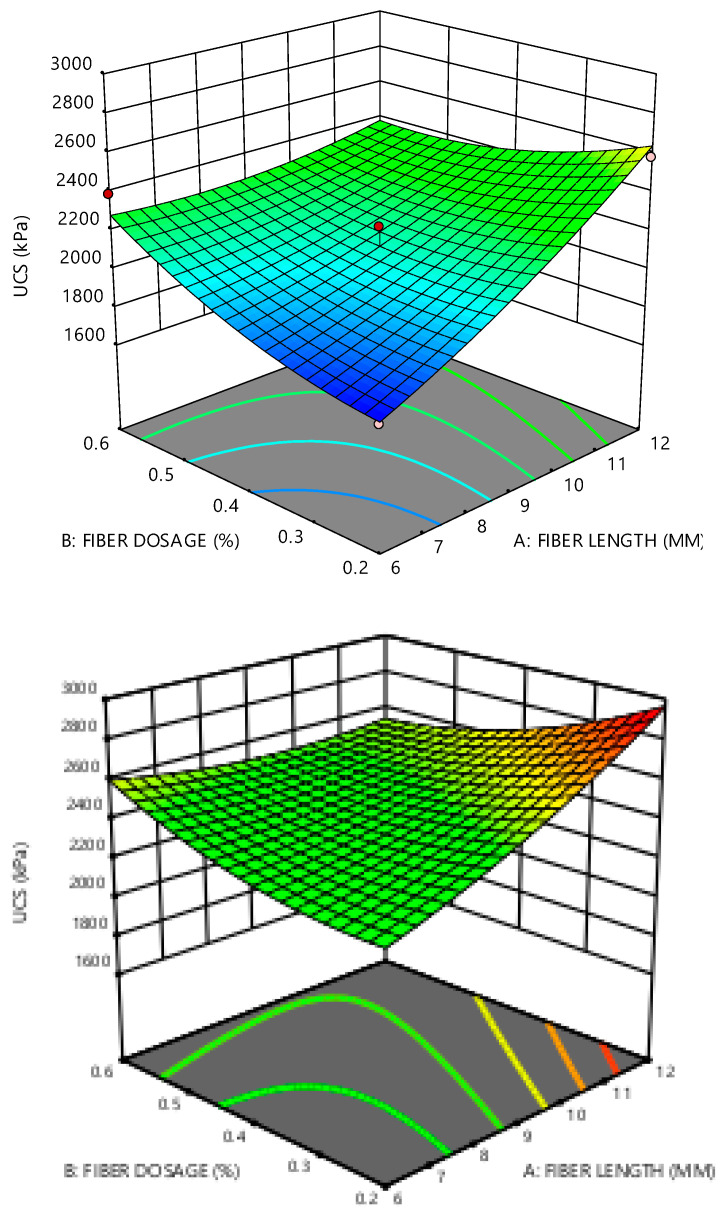

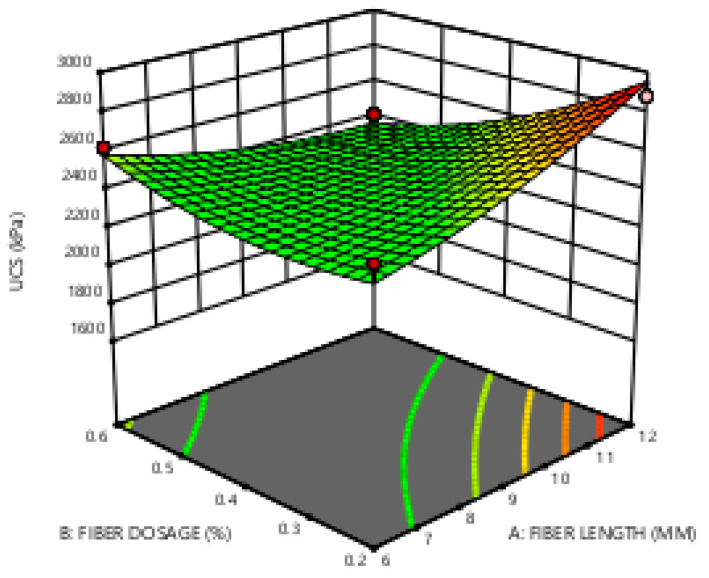
Three-dimensional plots showing the relationships between the response and the variable.

The effect of the parameters on the relationship between the responses and the variables is visualized by the 3D surface plots in Figure 8. One of the influential parameters is the FL considering the increase in the UCS with the increase in the FL.

### 4.3. Statistical Assessment of the Experimental Results of the HC

The influence of independent factors (i.e., FL, FD, and CT) and their interactions on the response variable (i.e., the HC value) was studied with ANOVA. As previously discussed, for the experimental results, the statistical significance of independent parameters (i.e., FL, FD, and CT) on the dependent variable (i.e., the HC value) at a 95% confidence level was analyzed using a full quadratic model. Statistically insignificant terms, with p greater than 0.05, were removed by performing backward analysis on the full quadratic equation. The percentage contributions to the response parameters in the model built were determined. ANOVA was performed. The results containing enumerated terms are shown in Table S3.

**Figure 9:**
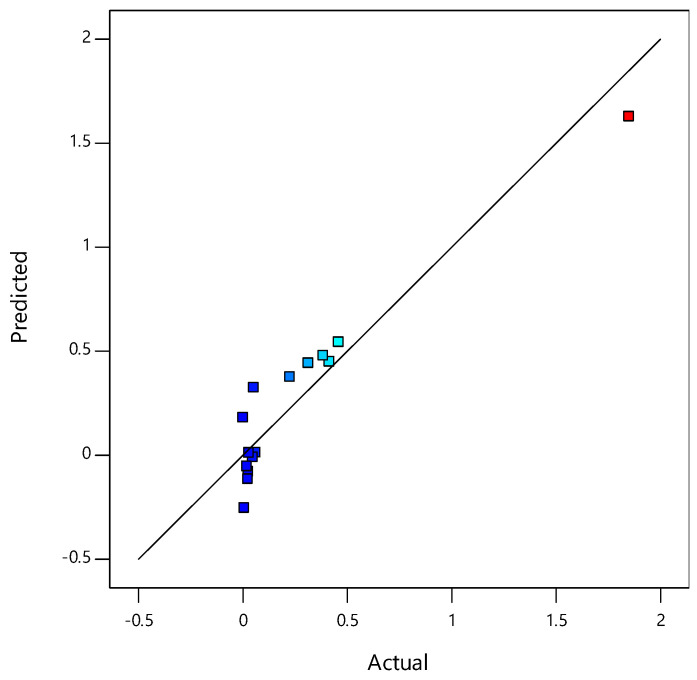
Predicted vs. actual values of the HC

The model F value of 99.82 suggests that the model was significant. Due to noise, there was only a 0.01% chance that an F value this large could occur. Values less than 0.0500 showed that the model terms were significant. A, B, C, AB, AC, BC, B^2^, and C^2^ were significant model terms in this case. Values greater than 0.1000 meant that the model terms were insignificant. The Pred R^2^ of 0.8820 was in suitable agreement with the Adj R^2^ of 0.8944 (i.e., the difference was less than 0.2). The signal-to-noise ratio was measured by model precision. Figure 9 shows that the prediction of the model equation was a statistically and moderately good match with the experimental results. The ratio of 39.268 represents an adequate signal.

HC = (6.24739 × (10^−6^)) + ((1.65974 × (10^−8^)) × A) − (0.000049 × B) − ((1.51641 × (10^−7^)) × C) + ((3.03021 × (10^−6^)) × (A × B)) − ((5.70034 × (10^−8^)) × (A × C)) − ((7.13123 × (10^−7^)) × (B × C)) + (0.000059 × B^2^) + ((2.05851 × (10^−8^)) × C^2^) (3)

**Figure 10:**
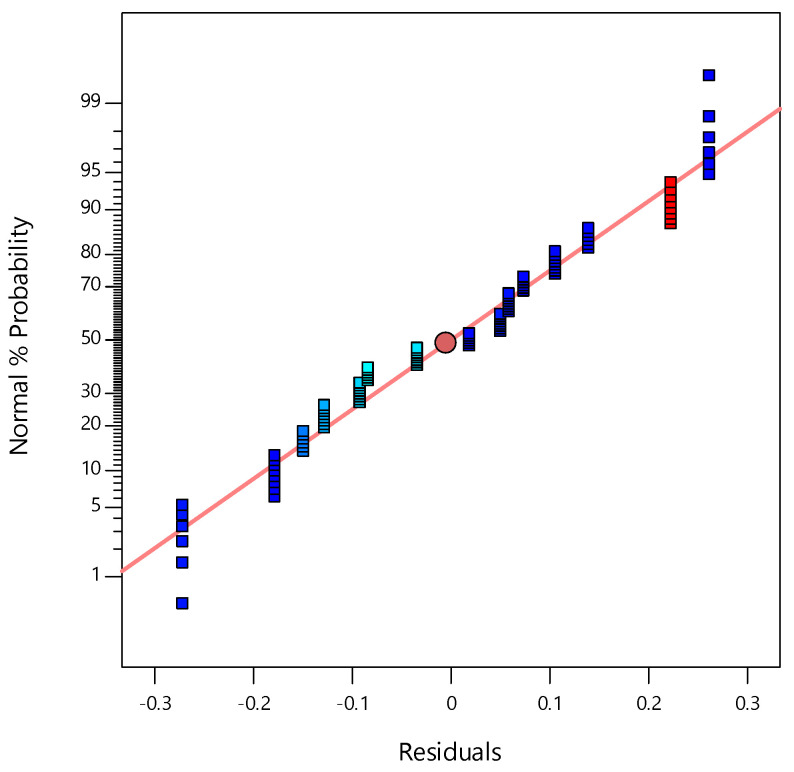
Average probability of the residuals for the HC

In Figure 10, the lower portion depicts the average probability of the residuals for the response, indicating that the experimental values were distributed relatively close to the straight line. A satisfactory correlation was found between these values.

**Figure 11:**
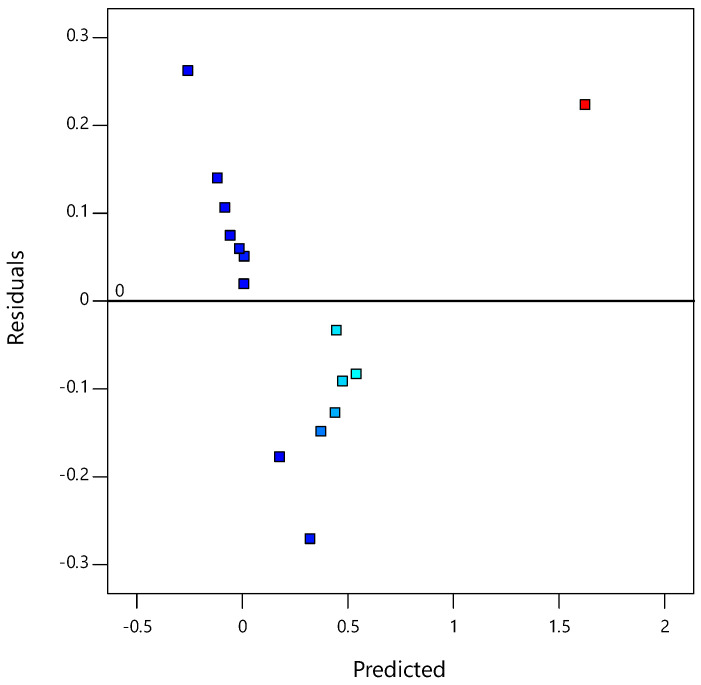
Plot of the residuals against the predicted response

The plot of the residuals against the predicted response verifies the constant variance assumption as seen from Figure 11. All points of the experimental runs were randomly distributed. All values lay mostly within the range of −2.00 × 10^−6^ to 2.00 × 10^−6^. These results show that the models proposed by the RSM were satisfactory. The contact variance assumptions were confirmed.

**Figure 12:**
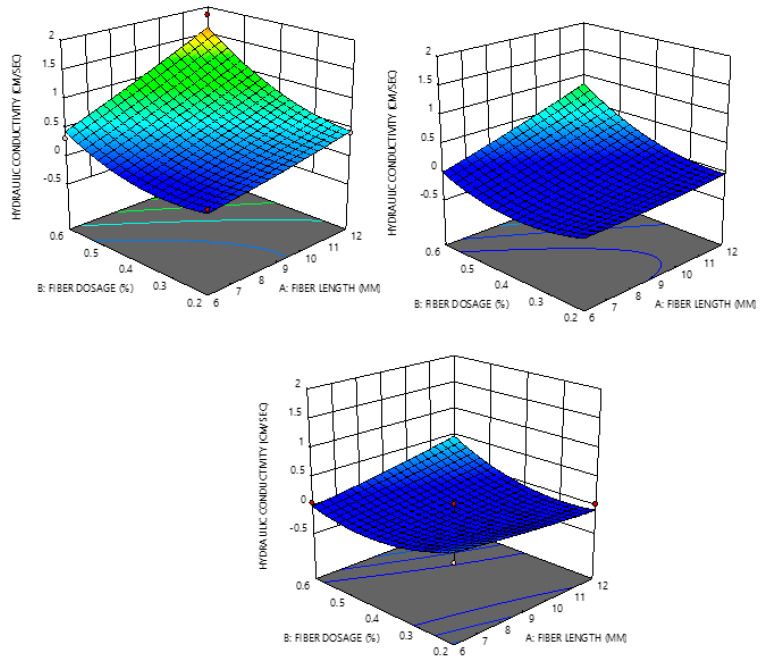
Three-dimensional plots showing the relationship between the responses and the independent parameters

The 3D plot visualizes the effect of the parameters on the relationship between the responses and the independent parameters. One of the influential parameters was the FL, as HC was found to increase with increase in FL as seen from Figure 12.

### 4.4. Optimization Results

The optimal proportions for the response variables (i.e., FL, FD, and CT) in the soil with lime and fiber mixture for each dependent response variable (i.e., CBR, UCS, and HC) were established by employing the approach of desirability functions (di). In this procedure, an individual desirability function (d_i_) ranging between 0 and 1 was converted from each dependent variable (y_i_). When the response variables (i.e., CBR, UCS, and HC) were outside of an appropriate area, the desirability function was equal to 0, while it was 1 if these variables were within an appropriate range, indicating that the proposed optimum values were statistically acceptable for the independent variables. The independent variables (i.e., FL, FD, and CT) of the mixture were chosen based on the highest values of the total desirability (D). The total desirability was determined as the geometrical mean.

(4) $$D={\left(d_{1}d_{2\;}d_{3}\cdots d_{m}\right)}_{m}^{1}\;$$

The ranges for the FL, FD, and CT variables in the optimization phase were selected according to the levels used for the experimental iterations for the CBR, UCS, and HC, as shown in Table 6, Table 7 and Table 8, with 0.2, 0.4, and 0.6% by soil dry weight for the FD; 6, 9, and 12 mm for the FL; and 0–14 days CT for the CBR, 60–360 days CT for the UCS, and 7–28 days CT for the HC. An optimization study was conducted to predict the optimum quantities of the FL, FD, and CT to achieve the required values of the geotechnical parameters based on the ranges given above. In Tables S4–S6, it can be seen that the CBR, UCS, and HC had optimum values with different candidate solutions that satisfied the ranges of variables mentioned above, utilizing the optimization approach presented throughout this study. Tables S4–S6 also show a total desirability (D) equal to 1, suggesting the ability of these candidate solutions to provide the right suitor values for training experimenters.

However, the best approach from all the effective ones for optimum values from Tables S4 and S5 is that with the independent parameters that render 11.15 mm for FL, 0.5% for FD, and 13.2 days for CT, as these yield a maximum value of 29.2% for the CBR, 11.7 for the FL, 0.3% for the FD, and 160 days for the CT, as well as a maximum value of 2655.579 kPa for UCS. The values obtained were exponential; thus, an alternate method for finding the right candidate for optimum values should be used. However, Table S6 shows that the best approach utilized independent variables yielding 10.455 mm for the FL, 0.491% for the FD, and 14.569 days for the CT, as these resulted in a minimum value of 5.17104 × 10^−7^ (cm/s) for the HC.

## 5. Conclusions

In this study response surface methodology analysis was employed to better understand the improvement in geotechnical properties such as unconfined compression strength, California bearing ratio, and hydraulic conductivity. The effect of fiber length and fiber dosage, along with curing time, in the presence of lime has been critically evaluated. The developed RSM model consists of full quadratic and backward analyses for predicting the optimum potential values of the input variables for the FD (0.2, 0.4, and 0.6% by soil dry weight), FL (6, 9, and 12 mm), and CT for the CBR, UCS, and HC. ANOVA was performed to determine the statistical significance of the RSM model and better understand the effects of the significant parameters (i.e., FL, FD, CT, FL*FD, FL*CT, FD*CT, FL2, FD2, and CT^2^) on the response variables of targeted geotechnical properties (i.e., the CBR, UCS, and HC). Curing periods of up to 14 days were considered for the CBR; 60–360 days for the UCS; and 7–28 days for HC, and these were used as performance indicators. The following conclusions can be drawn from the study.

- The experimental results and RSM analysis showed that the curing period had a considerable effect on the CBR and UCS data.
- Fiber dosage had a considerable effect on the HC data as, at higher fiber dosages, HC values increased significantly.
- RSM analysis revealed that lime-blended cases with an FL of 11.1 mm (~11 mm), 0.5% FD, and 13.2 (~13) days CT gave the maximum CBR value of 29.2%.
- For the UCS, an FL of 11.7 (~12 mm) mm, 0.3% FD, and 160 days CT gave the maximum value of 2656 kPa.
- For the HC, an FL of 10.5 mm, 0.5% FD, and 15 days CT gave the best HC value of 5.17 × 10^−7^ cm/s.

## Figures and Tables

**Table 1:** Testing procedures adopted.

Property	Value for Untreated Soil (Without Lime and Fiber)	Relevant Code
Liquid limit, plastic limit and plasticity Index	66%, 32%, and 34%	ASTM D4318 [26]
Specific gravity	2.85	ASTM D854 [27]
Bar linear shrinkage test	31%	Tex-107-E [28]
Unconfined compression strength test	598.11 kPa	ASTM D2166 [29]
One-dimensional fixed ring oedometer Consolidation test	0.109 (compression index) and 0.069 (swell index)	ASTM D2435 [30]
Hydraulic conductivity	6.77 × 10^−7^ (cm/s)	ASTM D5084 [31]
California bearing ratio test	5.96	ASTM D1883 [32]

**Table 2:** Experimental California bearing ratio (CBR) values under the soaked condition.

Fiber Length (mm)	Fiber Dosage (%)	Curing Time (days)	CBR (%)
6	0.2	0	11
6	0.4	0	11.7
6	0.6	0	12.5
6	0.2	7	16.3
6	0.4	7	17.7
6	0.6	7	19.1
6	0.2	14	24.6
6	0.4	14	27.1
6	0.6	14	29.7
9	0.2	0	11.4
9	0.4	0	12.1
9	0.6	0	12.9
9	0.2	7	17.2
9	0.4	7	18.5
9	0.6	7	19.9
9	0.2	14	26.2
9	0.4	14	28.5
9	0.6	14	30.8
12	0.2	0	11.8
12	0.4	0	12.6
12	0.6	0	13.3
12	0.2	7	18.1
12	0.4	7	19.3
12	0.6	7	20.6
12	0.2	14	27.9
12	0.4	14	29.9
12	0.6	14	31.8

**Table 3:** Experimental values of the unconfined compression strength (UCS).

Fiber Length (MM)	Fiber Dosage (%)	Curing Time (days)	UCS (kPa)
6	0.2	60	1767
6	0.4	60	2070
6	0.6	60	2390
6	0.2	210	1996
6	0.4	210	1998
6	0.6	210	2110
6	0.2	360	2505
6	0.4	360	1912
6	0.6	360	2620
12	0.2	60	2579
12	0.4	60	2412
12	0.6	60	2287
12	0.2	210	3171
12	0.4	210	2912
12	0.6	210	2673
12	0.2	360	2881
12	0.4	360	2589
12	0.6	360	2412

**Table 4:** Experimental values of the hydraulic conductivity (HC).

Fiber Length (mm)	Fiber Dosage (%)	Curing Time (days)	Hydraulic Conductivity (cm/s)
6	0.2	7	6.21 × 10^−7^
6	0.4	7	8.23 × 10^−7^
6	0.6	7	3.14 × 10^−6^
6	0.2	17	2.55 × 10^−8^
6	0.4	17	6.72 × 10^−8^
6	0.6	17	8.27 × 10^−7^
6	0.2	28	1.69 × 10^−8^
6	0.4	28	5.63 × 10^−8^
6	0.6	28	4.77 × 10^−7^
12	0.2	7	4.15 × 10^−6^
12	0.4	7	3.71 × 10^−6^
12	0.6	7	1.85 × 10^−5^
12	0.2	17	3.66 × 10^−7^
12	0.4	17	5.28 × 10^−7^
12	0.6	17	8.37 × 10^−6^
12	0.2	28	2.63 × 10^−7^
12	0.4	28	4.22 × 10^−7^
12	0.6	28	3.86 × 10^−6^

**Table 5:** Experimental levels of each variable.

Variables	Level		
	−1	0	1
Fiber length	6 mm	9 mm	12 mm
Fiber dosage	0.2%	0.4%	0.6%
CBR curing time	0 days	7 days	14 days
UCS curing time	60 days	210 days	360 days
HC curing time	7 days	17 days	28 days

**Table 6:** Face-centered central composite design (FCCCD) experiment values and CBR results.

Design			Program			CBR (%)	
FL	FD	CT	FL(mm)	FD (%)	CT (Days)	Actual	RSM
−1	−1	−1	6	0.2	0	11	10.88
−1	1	−1	6	0.6	0	12.5	12.63
−1	−1	1	6	0.2	14	24.6	24.78
−1	1	1	6	0.6	14	29.7	29.54
−1	0	0	6	0.4	7	17.7	17.63
0	−1	0	9	0.2	7	17.2	17.07
0	1	0	9	0.6	7	19.9	20.024
0	0	−1	9	0.4	0	12.1	12.11
0	0	1	9	0.4	14	28.5	28.47
1	−1	−1	12	0.2	0	11.8	11.95
1	1	−1	12	0.6	0	13.3	13.11
1	−1	1	12	0.2	14	27.9	27.76
1	1	1	12	0.6	14	31.8	31.91
1	0	0	12	0.4	7	19.3	19.36

**Table 7:** FCCCD experiment values and UCS results.

Design			Program			UCS (kPa)	
FL	FD	CT	FL(mm)	FD (%)	CT (Days)	Actual	RSM
−1	−1	1	6	0.2	60	1766.17	1781.52
−1	1	1	6	0.6	60	2390.65	2285.8
−1	−1	−1	6	0.2	360	2505.39	2414.71
−1	1	−1	6	0.6	360	2620.12	2581.46
−1	0	0	6	0.4	210	1998.26	2290.24
0	−1	0	9	0.2	210	2582.61	2503.18
0	1	0	9	0.6	210	2391.21	2493.61
0	0	1	9	0.4	60	2223.01	2125.13
0	0	0	9	0.4	210	2453.82	2407.94
1	−1	1	12	0.2	60	2579.15	2641.42
1	1	1	12	0.6	60	2287.39	2388.04
1	−1	−1	12	0.2	360	2880.73	2953.50
1	1	−1	12	0.6	360	2411.96	2362.59
1	0	0	12	0.4	210	2911.87	2642.87

**Table 8:** FCCCD experiment values and HC results.

Design			Program			HC (cm/s)	
FL	FD	CT	FL(mm)	FD (%)	CT (Days)	Actual	RSM
−1	−1	1	6	0.2	7	6.21 × 10^−7^	1.47 × 10^−7^
1	−1	1	12	0.2	7	4.15 × 10^−6^	4.64 × 10^−6^
−1	1	1	6	0.6	7	3.14 × 10^−6^	4.7 × 10^−6^
1	1	1	12	0.6	7	1.85 × 10^−5^	1.65 × 10^−5^
−1	−1	−1	6	0.2	28	1.69 × 10^−8^	1.92 × 10^−6^
1	−1	−1	12	0.2	28	2.63 × 10^−8^	−7.8 × 10^−7^
−1	1	−1	6	0.6	28	4.77 × 10^−7^	4.81 × 10^−7^
1	1	−1	12	0.6	28	3.86 × 10^−6^	5.06 × 10^−6^
−1	0	0	6	0.4	17	6.72 × 10^−8^	−2.4 × 10^−6^
1	0	0	12	0.4	17	5.28 × 10^−7^	3.37 × 10^−6^
0	−1	0	9	0.2	17	1.95 × 10^−7^	−4.9 × 10^−7^
0	1	0	9	0.6	17	4.59 × 10^−6^	5.7 × 10^−6^
0	0	1	9	0.4	7	2.26 × 10^−6^	3.87 × 10^−6^
0	0	−1	9	0.4	28	2.39 × 10^−7^	−9.5 × 10^−7^
0	0	0	9	0.4	17	2.97 × 10^−7^	2.43 × 10^−7^

## Data Availability

Data is contained within the article.

## Supplementary Materials

The following are available online at https://www.mdpi.com/1996-1944/14/6/1535/s1, Table S1: ANOVA for Quadratic model for CBR; Table S2: ANOVA for Quadratic model for UCS; Table S3: ANOVA for Quadratic model for HC; Table S4: Optimized Results of CBR; Table S5: Optimized Results of UCS; Table S6: Optimized Results of HC.
