# Supplementary material for: Response Surface Method Analysis of Chemically Stabilized Fiber-Reinforced Soil
Source: Materials (Basel). 2021 Mar 21;14(6):1535. doi: 10.3390/ma14061535 (PMC8003954; doi:10.3390/ma14061535)
Supplement: Supplementary file 1 [file materials-14-01535-s001.pdf]

materials

MDPI

Supplementary Materials: Response Surface Method Analysis of Chemically Stabilized Fiber-Reinforced Soil

Abdullah Almajed [1,*], Dinesh Srirama [2] and Arif Ali Baig Moghal [2]

[1] Department of Civil Engineering, College of Civil Engineering, King Saud University, Riyadh 11421, Saudi Arabia
[2] Department of Civil Engineering, National Institute of Technology, Warangal 506004, Telangana State, India; dinesh1991@student.nitw.ac.in (D.S.); baig@nitw.ac.in or reach2arif@gmail.com (A.A.B.M.)
* Correspondence: alabduallah@ksu.edu.sa

Table S1. ANOVA for Quadratic model for CBR.

Source	Sum of Squares	Degree of Freedom	Mean Square	F-value	p-value	
Model	5427.98	9	603.11	33444.15	< 0.0001	significant
A-Fiber Length	56.62	1	56.62	3139.92	< 0.0001	
B-Fiber Dosage	165.64	1	165.64	9185.38	< 0.0001	
C-Curing Time	5084.03	1	5084.03	2.819×10^5	< 0.0001	
AB	1.44	1	1.44	79.85	< 0.0001	
AC	14.44	1	14.44	800.74	< 0.0001	
BC	36.00	1	36.00	1996.31	< 0.0001	
C^2	50.17	1	50.17	2782.12	< 0.0001	
Residual	1.73	96	0.0180			
Lack of Fit	1.73	5	0.3462			
Pure Error	0.0000	91	0.0000			
Cor Total	5429.71	105				
R^2	0.9997		Adjusted R^2	0.9997		
Predicted R^2	0.9996		Adeq Precision	510.0302		
Standard Deviation	0.1343		Coefficient of Variance %	0.6778		
Mean	19.81					

Factor coded.
The Sum of squares is Type III–Partial.

Table S2. ANOVA for Quadratic model for UCS.

Source	Sum of Squares	Degree of Freedom	Mean Square	F-value	p-value	
Model	7.313×10^{6}	9	8.126×10^{5}	50.36	< 0.0001	significant
A-Fiber Length	1.950×10^{6}	1	1.950×10^{6}	120.82	< 0.0001	
B-Fiber Dosage	35607.83	1	35607.83	2.21	0.1407	
C-Curing Time	1.754×10^{6}	1	1.754×10^{6}	108.68	< 0.0001	
AB	2.296×10^{6}	1	2.296×10^{6}	142.30	< 0.0001	
AC	4.151×10^{5}	1	4.151×10^{5}	25.72	< 0.0001	
BC	4.586×10^{5}	1	4.586×10^{5}	28.42	< 0.0001	
A^2	53866.53	1	53866.53	3.34	0.0708	
B^2	1.283×10^{5}	1	1.283×10^{5}	7.95	0.0058	
C^2	4.045×10^{5}	1	4.045×10^{5}	25.07	< 0.0001	
Residual	1.549×10^{6}	96	16135.73			
Lack of Fit	1.535×10^{6}	5	3.071×10^{5}	2057.92	< 0.0001	significant
Pure Error	13579.37	91	149.22			
Cor Total	8.862×10^{6}	105				
R^2	0.8252		Adjusted R^2	0.8088		
Predicted R^2	0.7881		Adeq Precision	30.0369		
Standard Deviation	127.03		Coefficient of Variance %	5.25		
Mean	2420.96					

Factor coding is Coded.
Sum of squares is Type III-Partial.

Table S3. ANOVA for Quadratic model for HC.

Source	Sum of Squares	Degree of Freedom	Mean Square	F-value	p-value	
Model	2.169×10^{-9}	9	2.410×10^{-10}	99.82	< 0.0001	significant
A-Fiber Length	3.896×10^{-10}	1	3.896×10^{-10}	161.34	< 0.0001	
B-Fiber Dosage	4.793×10^{-10}	1	4.793×10^{-10}	198.51	< 0.0001	
C-Curing Time	4.411×10^{-10}	1	4.411×10^{-10}	182.69	< 0.0001	
AB	2.116×10^{-10}	1	2.116×10^{-10}	87.62	< 0.0001	
AC	2.100×10^{-10}	1	2.100×10^{-10}	86.96	< 0.0001	
BC	1.460×10^{-10}	1	1.460×10^{-10}	60.49	< 0.0001	
A^2	1.079×10^{-12}	1	1.079×10^{-12}	0.4471	0.5053	
B^2	8.712×10^{-11}	1	8.712×10^{-11}	36.08	< 0.0001	
C^2	6.238×10^{-11}	1	6.238×10^{-11}	25.83	< 0.0001	
Residual	2.318×10^{-10}	96	2.415×10^{-12}			
Lack of Fit	2.218×10^{-10}	5	4.436×10^{-11}	403.08	< 0.0001	significant
Pure Error	1.001×10^{-11}	91	1.100×10^{-13}			
Corr. Total	2.401×10^{-9}	105				
R^2	0.9035		Adjusted R^2	0.8944		
Predicted R^2	0.8820		Adeq Precision	39.2676		
Standard Deviation	1.554×10^{-6}		Coefficient of Variance %	54.78		
Mean	2.836×10^{-6}					

Factor coding is Coded.
Sum of squares is Type III-Partial.

Table S4. Optimized Results of CBR.

Fiber Length	Fiber Dosage	Curing Time	CBR %	RSM CBR(%)	Desirability
6	0.6	14	29.7	29.238	1
6.484	0.28	11.135	21.36	21.759	1
6.588	0.531	9.278	21.44	21.539	1
6.635	0.399	5.253	15.65	15.876	1
6.715	0.528	9.752	22.52	22.201	1
6.799	0.522	8.184	20.26	20.098	1
6.922	0.537	2.89	14.64	14.492	1
7.147	0.533	8.808	21.05	21.108	1
7.61	0.499	9.805	22.77	22.334	1
7.853	0.448	4.473	15.39	15.712	1
8.918	0.325	3.978	14.58	14.859	1
8.967	0.436	12.441	26.65	26.22	1
9.088	0.298	13.274	26.13	26.245	1
9.22	0.553	9.43	22.32	22.787	1
9.367	0.514	8.293	20.69	20.972	1
9.487	0.206	5.344	15.13	15.668	1
9.545	0.585	2.666	14.86	14.959	1
9.618	0.542	8.143	21.24	21.051	1
9.807	0.516	11.813	26.25	26.301	1
10.759	0.21	11.155	23.38	23.052	1
10.918	0.217	4.506	15.49	15.376	1
11.149	0.512	13.237	29.43	29.186	1
11.163	0.517	9.74	23.76	23.617	1
11.293	0.397	13.768	29.11	29.073	1
11.361	0.272	3.73	15.18	15.036	1
11.647	0.454	2.701	14.92	14.864	1
11.713	0.532	7.75	21.16	21.071	1
11.752	0.356	10.366	23.71	23.524	1
11.862	0.299	10.756	23.59	23.689	1
11.897	0.263	10.284	22.45	22.752	1
11.951	0.219	4.216	15.32	15.421	1

Table S5. Optimized Results of UCS.

Fiber Length	Fiber Dosage	Curing Time	UCS (kPa)	RSM UCS (kPa)	Desirability
11.714	0.335	159.746	2655.566	2655.579	1
6.764	0.584	86.915	2321.274	2321.277	1
11.069	0.276	293.566	2743.815	2743.857	1
11.894	0.391	343.168	2600.328	2600.385	1
7.25	0.587	234.906	2548.533	2548.56	1
6.119	0.52	187.441	2443.413	2443.43	1
7.348	0.264	273.374	2437.136	2437.172	1
11.53	0.573	146.623	2497.893	2497.904	1
6.57	0.37	220.629	2351.381	2351.405	1
9.464	0.386	242.227	2498.651	2498.68	1
9.647	0.491	254.949	2486.348	2486.38	1

Table S6. Optimized Results of HC.

Fiber Length	Fiber Dosage	Curing Time	HC (cm/sec)	RSM HC (cm/sec)	Desirability
11.4606	0.437	22.467	3.15×10^{-6}	-3.22735×10^{-6}	1
6.487	0.574	23.112	1.06×10^{-6}	-1.56453×10^{-6}	1
9.568	0.558	7.240	6.45×10^{-6}	6.76421×10^{-6}	1
9.645	0.264	25.496	4.11×10^{-6}	-4.00331×10^{-6}	1
7.389	0.540	16.642	1.21×10^{-6}	-1.03548×10^{-6}	1
6.215	0.497	23.464	2.78×10^{-6}	-2.92273×10^{-6}	1
6.000	0.350	21.194	3.54×10^{-6}	-3.71809×10^{-6}	1
10.455	0.491	14.569	5.26×10^{-6}	5.17104×10^{-7}	1
8.339	0.528	10.255	2.81×10^{-6}	2.17776×10^{-6}	1
11.744	0.351	23.715	4.75×10^{-6}	-4.82792×10^{-6}	1
10.402	0.447	19.116	2.39×10^{-6}	-2.40957×10^{-6}	1
